# Antifatigue effect of *Gracilaria eucheumoides* in mice

**DOI:** 10.3892/etm.2013.1346

**Published:** 2013-10-15

**Authors:** JIN-TING SHAO, MEI-YAN WANG, LU-BIN ZHENG

**Affiliations:** 1 Physical and Military Training Department, Zhejiang University of Finance and Economics, Hangzhou, Zhejiang 310018, P.R. China; 2 Sports Center, Zhejiang University of Finance and Economics Dongfang College, Hangzhou, Zhejiang 314408, P.R. China; 3 Physical Education Department, Shanghai University of Sport, Shanghai 200438, P.R. China

**Keywords:** seaweed, physical symptoms, glucose transport protein 4, AMP-activated protein kinase

## Abstract

*Gracilaria eucheumoides* Linn (Gracilariaceae; *G. eucheumoides*) is abundant in dietary fiber, which aids the clearance of excess cholesterol from the blood and maintains stable blood glucose levels. The aim of the present study was to investigate the antifatigue effect of *G. eucheumoides* in mice and the physiological and molecular mechanisms underlying this effect. Mice were randomly divided into four groups and three of the groups were administered different doses of *G. eucheumoides* extract. A loaded swimming test demonstrated that the swimming times of the low-, medium- and high-dose groups were longer than those of the control group. Examinations revealed that the liver and muscle glycogen, lactate dehydrogenase and blood glucose concentration levels of the treatment groups were higher than those of the control group (P<0.05). However, this was not the case for lactic acid concentration (P>0.05). Quantitative polymerase chain reaction showed that the gene expression levels of glucose transport protein 4 and AMP-activated protein kinase in the medium-dose group exhibited the largest increases, compared with the other treatment groups, and were 3.0- and 1.8-fold higher than those in the control group, respectively. The results of the present study indicated that *G. eucheumoides* exerts an antifatigue effect on mice.

## Introduction

Marine algae, also known as marine vegetables, are naturally rich in polysaccharides, minerals, polyunsaturated fatty acids, vitamins and bioactive molecules. Their nutritional value is markedly higher than that of terrestrial vegetables. The bioactive compounds in marine algae have been reported to possess strong antihypertensive (1–3), antitumor (4,5), anti-inflammatory (6–8), antidiabetic (9,10) and anticoagulant (11,12) properties. In addition, the prebiotic health potential of polysaccharides from seaweeds has been increasingly studied in recent years.

Fatigue, a phenomenon of decreased efficiency following continuous study or work, may be divided into mental and physical fatigue. It mainly manifests as a physical decrease in muscle tone and exercise tolerance due to an accumulation of lactic acid (LC) and other metabolites (13). Fatigue also leads to yawning, as a result of a build-up of carbon dioxide that stimulates the respiratory center. At present, work and life stresses are escalating with the increasing pace of life. Thus, fatigue is common and may significantly affect daily routines. However, a systematic and authoritative hypothesis to explain the induction of fatigue has yet to be suggested. As technology has progressed, a number of scientists have begun to investigate fatigue-associated genes and proteins in order to reveal the mechanisms underlying fatigue and improve therapeutic strategies (14,15).

*Gracilaria eucheumoides* Linn (Gracilariaceae; *G. eucheumoides*), a species of seaweed, is a type of natural edible green algae that primarily grows on the southeast coast of China (16). It contains numerous vitamins and microelements, and is abundant in dietary fiber, which aids the clearance of excess cholesterol from the blood and maintains stable blood glucose levels (17). Therefore, *G. eucheumoides* may have medicinal value and has the potential to be exploited as a functional ingredient in human and animal health applications. Although *G. eucheumoides* has previously been used in health products in China, to the best of our knowledge its antifatigue effects have not yet been reported. In the present study, the water extract of *G. eucheumoides* was administered to three groups of mice, and the exhaustive swimming times and relevant physiological indices were measured in order to determine the antifatigue effects of *G. eucheumoides*. Furthermore, the expression levels of glucose transport protein 4 (GLUT4) and AMP-activated protein kinase (AMPK) were analyzed using quantitative polymerase chain reaction (qPCR) and western blotting. The results demonstrated that *G. eucheumoides* extract may improve the ability to fight fatigue in mice.

## Materials and methods

### Preparation of G. eucheumoides extract

The water extract of *G. eucheumoides* was prepared following a method described previously (18).

### Grouping and treatment of the mice

Healthy male Kunming mice, weighing 10–22 g, were purchased from the Institute of Laboratory Animal Sciences of the Chinese Academy of Medical Sciences (Beijing, China). A total of 40 mice were randomly divided into four groups of ten: Low- (20 mg/kg), medium- (40 mg/kg) and high-dose (80 mg/kg) groups and a normal control group (normal saline). Each mouse was weighed and fed with 0.02 ml/g *G. eucheumoides* extract by gavage daily for 30 days. The *G. eucheumoides* extract was diluted with purified water to the designated concentration. The health status of the mice was observed each day and all the animals were weighed every three days. The quantity of feed was determined in line with the weight of the animals for the 30 days. All mice were freely fed and watered during the experiment. All animal procedures were reviewed and approved by the Shanghai University of Sport Science Research Ethics Committee (chiCTR-TRC-120050028; Shanghai, China).

### Loaded swimming test

Thirty minutes subsequent to the final administration of *G. eucheumoides*, a lead sheath, weighing 5% of the body weight of the mouse, was tied to the root of the tail, prior to the mouse being placed in a water container. The swimming time (time between being placed in the water and sinking underwater for >10 sec) was recorded. The water depth was ≥30 cm and the temperature was 25±1°C.

### Determination of hepatic and muscle glycogen

Thirty minutes subsequent to the final feed, non-loaded mice were placed in a water container and left to swim for 90 min at a temperature of 25±1°C. The mice were then sacrificed by cervical vertebra dislocation, cleaned using normal saline and dried with filter paper. Subsequently, 100 mg liver tissue and 500 mg quadriceps femoris tissue were weighed and diluted to 10% homogenate with normal saline. Following centrifugation at 800–1,800 × g for 10 min, the supernatant was used to determine the quantity of glycogen using the Anthrone colorimetric method.

### Measurement of LC, lactate dehydrogenase (LDH) and blood glucose concentrations

Thirty minutes subsequent to the final feed, non-loaded mice were left to swim for 90 min at a temperature of 25±1°C. Subsequently, venous blood was collected from the tail and the LC, LDH and blood glucose concentrations were determined using the LC kit (Cat. No. A019-1), LDH kit (Cat. No.A020) and glucose kit (Cat. No.F006) from Nanjing Jiancheng BioEngineering Institute (Nanjing, China), in accordance with the manufacturer’s instructions.

### qPCR

Thirty minutes subsequent to the final feed, non-loaded mice were left to swim for 90 min at a temperature of 25±1°C. Subsequently, the mice were sacrificed by cervical vertebra dislocation and cleaned using normal saline. Following drying with filter paper, 50–100 mg quadriceps femoris tissue was weighed and RNA was isolated using TRIzol reagent^®^ (Invitrogen Life Technologies, Carlsbad, CA, USA) according to the manufacturer’s instructions. The specific primer pairs designed for the amplification of GLUT4 and AMPK, and the reference gene of 18s RNA, are exhibited in Table I. All qPCR reactions were performed using a CFX-96 Real-Time PCR detection System (Bio-Rad, Hercules, CA, USA). Each 25-μl reaction consisted of 12.5 μl SYBR Green I, 5 μl cDNA, 2.5 μl forward and reverse primer, and 2.5 μl sterile water. The conditions for PCR were 40 cycles of: 95°C, 3 sec; 60°C, 5 sec; and 72°C, 30 sec. Following PCR, a melting curve analysis was performed in order to demonstrate the specificity of the PCR products. Each reaction was run in triplicate and the results were analyzed using SPSS 15.0 statistical software (SPSS, Inc., Chicago, IL, USA).

### Protein extraction and western blot analysis

Thirty minutes subsequent to the final feed, non-loaded mice were left to swim for 90 min at a temperature of 25±1°C. Subsequently, the mice were sacrificed by cervical vertebra dislocation and cleaned using physiological saline. Following drying with filter paper, 40 mg quadriceps femoris tissue was weighed and lysed in a lysis buffer consisting of 50 mM Tris-HCl (pH 7.4–8.0), 150 mM NaCl, 5 mM EDTA, 1% Triton™ X-100, and 1 mM PMSF. Protein concentrations were determined using a bicinchoninic acid protein assay (Pierce Chemical Co., Rockford, IL, USA) using bovine serum albumin as a standard. Protein samples of a total of 20 μg were separated using sodium dodecyl sulfate-polyacrylamide gel electrophoresis (SDS-PAGE) technology and were transferred to polyvinylidene difluoride (PVDF) membranes. The plates were blocked with 5% (w/v) nonfat milk powder for 2 h at room temperature and immunoblotted with primary antibodies. Following one night at 4°C, the secondary antibodies were added and the antigen-antibody complex was detected using an enhanced chemiluminescence (ECL) kit (Amersham Pharmacia Biotech, Piscataway, NJ, USA) in accordance with the manufacturer’s instructions. β-actin was treated as a control. The primary antibodies and secondary antibodies used were as follows: goat anti-GLUT4 (Santa Cruz Biotechnology, Inc., Santa Cruz, CA, USA; sc-1608), goat anti-AMPK (Santa Cruz Biotechnology, Inc.; sc-19126) and donkey anti-goat IgG-HRP (Santa Cruz Biotechnology, Inc.; sc-2020).

### Statistical analysis

All statistical procedures were performed using SPSS version 15.0 (SPSS, Inc.). Data are expressed as the mean ± standard deviation (SD). Differences between groups were analyzed using one-way analysis of covariance. P<0.05 was considered to indicate a statistically significant difference and P<0.01 was considered highly significant.

## Results

### Effect of G. eucheumoides extract on the loaded swimming time of mice

The mice in the three *G. eucheumoides* extract treatment groups were subjected to the loaded swimming test. The results of the test are exhibited in Table II.

The loaded swimming times of the low-, medium- and high-dose groups were greater than those of the control group. Among the three treatment groups, the loaded swimming time of the medium-dose group was the longest (41.3±0.5 min). The increment rate of the loaded swimming time for the medium-dose group was 32%, which was significantly different from the control group. Furthermore, the increment rates of the low- and high-dose groups showed significant increases of 19.1 and 16.3%, respectively, compared with the control group. These results indicated that *G. eucheumoides* extract may extend the loaded swimming time of mice.

### Effect of G. eucheumoides extract on liver and muscle glycogen, LC, LDH and blood glucose concentrations of mice

In order to investigate the mechanism underlying the antifatigue effect of *G. eucheumoides* extract, the liver and muscle glycogen, LC, LDH and blood glucose concentrations of mice were measured following the non-loaded swimming test (Table III).

It was revealed that the relevant physiological indices of the treatment groups were significantly increased compared with those of the control group (P<0.05), with the exception of the LC concentration. In the treatment groups, liver glycogen and muscle glycogen and blood glucose concentrations initially increased and then decreased as the extract dose was increased. LDH concentration increased with an increasing dose, while LC concentration decreased. These results indicated that *G. eucheumoides* extract significantly alters the liver and muscle glycogen, LDH, LC and blood glucose concentrations of mice.

### Effect of G. eucheumoides extract on the gene expression levels of GLUT4 and AMPK

**Figure 1:**
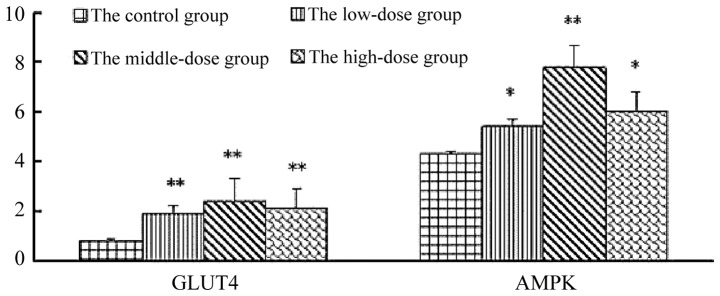
Expression of GLUT4 and AMPK genes in mice fed with *G. eucheumoides* extract of different concentrations.^*^Difference between the treatment and control groups was significant (P<0.05); ^**^difference between the treatment and control groups was highly significant (P<0.01). GLUT4, glucose transport protein 4; AMPK, AMP-activated protein kinase.

To investigate the molecular role of *G. eucheumoides* extract in mice, the expression levels of GLUT4 and AMPK were determined using qPCR (Fig. 1). Compared with the control group, the expression levels of GLUT4 and AMPK were significantly increased in the treatment groups. The expression levels of the two genes were increased to the greatest extent in the medium-dose group and were 3.0- and 1.8-fold higher than those in the control group, for GLUT4 and AMPK respectively. These results demonstrated that *G. eucheumoides* extract may regulate the expression levels of GLUT4 and AMPK in the muscle of mice.

### Effect of G. eucheumoides extract on protein expression levels of GLUT4 and AMPK

**Figure 2:**
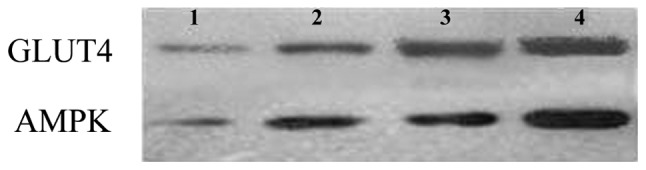
GLUT4 and AMPK protein expression in mice fed with *G. eucheumoides* extract of different concentrations. Lane 1, control group; 2, low-dose group; 3, medium-dose group; 4, high-dose group. GLUT4, glucose transport protein 4; AMPK, AMP-activated protein kinase.

The protein expression levels of GLUT4 and AMPK were further characterized by western blotting (Fig. 2). The results showed that protein expression levels of GLUT4 and AMPK increased gradually with an increasing extract dose. Although the expression levels of these proteins reached their peaks in the high-dose group, the increasing trends were different between the proteins. GLUT4 was significantly increased from the low-dose group to the medium-dose group, while AMPK was significantly increased from the medium-dose group to the high-dose group. In combination, these results suggested that *G. eucheumoides* extract enhanced the protein expression of GLUT4 and AMPK in mice.

## Discussion

Fatigue is a normal physiological or psychological phenomenon, usually associated with physical and/or mental weakness, varying from a general state of lethargy to a specific work-induced burning sensation within the muscles. Prolonged and high intensity exercise leads to an inadequate oxygen supply at the physiological level. In order to maintain physical functioning, the body primarily performs glucose metabolism, which is dependent on the glycolytic pathway and results in significant consumption of glycogen and accumulation of fatigue-inducing metabolites, including LC (13).

The bioactive compounds derived from seaweed may be classified into two types: Indigestible intercellular viscous polysaccharides, including alginic acid, fucoidan, rhodophyte agar and carrageenan (19,20), and digestible compounds with low molecular weights, including halide, terpenoid, bromophenol compound, algae tannin and laminin (5,21,22). The latter type are capable of being absorbed by the human body and regulate metabolism directly and indirectly. *G. eucheumoides* contains several types of vitamins and microelements, and is abundant in dietary fiber. The human consumption of dietary fiber has been correlated with a number of health-promoting effects, including growth promotion, the protection of beneficial intestinal flora and the reduction of the overall glycemic response. Thus, at present *G. eucheumoides* is is a topic of interest.

In the present study, the loaded swimming times of mice fed with *G. eucheumoides* extract were significantly increased compared with those of the control group, indicating that the extract may enhance tolerance to fatigue in mice. Analysis of physical indices demonstrated that the liver and muscle glycogen, LDH and blood glucose concentrations of mice in the treatment groups were significantly increased compared with those in the control group. Furthermore, the LC concentrations of the treatment groups were significantly decreased compared with those of the control group. Glycogen serves as a form of energy storage in animals and is stored primarily in the cells of the liver and the muscles (23). Energy is supplied via hepatic glycogen degradation when blood glucose is depleted. The results of this study suggested that G. eucheumoides may improve the energy efficiency of mice.

During high intensity exercises, blood glucose is metabolized and oxidized to pyruvate, and LC is produced from the pyruvate at a greater speed than tissues are capable of removing it. Therefore, the LC concentration begins to rise (24,25). The LC concentration of mammalian blood is 1–2 mM when sitting still and may increase to 20 mM during strenuous exercise. During the present experiments, the LC concentration decreased to 11.3 mM in the high-dose group and the concentration of LDH increased following exercise. LDH catalyzes the oxidation of LC, forming pyruvate. Therefore, mice fed with *G. eucheumoides* extract experienced a reduction in blood LC concentration as a result of increased LDH concentration.

In order to investigate the molecular mechanisms underlying the antifatigue effect of *G. eucheumoides*, the mRNA and protein expression of AMPK and GLUT4 were examined using qPCR and western blotting, respectively. AMPK has a central role in the control of metabolism in cells, and operates an alternative pathway of energy synthesis when cellular energy is low (26,27). GLUT4 transports glucose in adipose tissue and muscle, and is important in energy metabolism and the antifatigue process (28). In this study, mRNA and protein expression levels of AMPK and GLUT4 were significantly increased in the treatment groups compared with those of the control group. However, the expression levels were increased in different manners. The mRNA and protein levels of GLUT4 increased to their highest levels in the high-dose group. The mRNA expression of AMPK also increased to its highest level in the medium-dose group; however, the protein expression level of AMPK increased to its highest level in the high-dose group. It is possible that *G. eucheumoides* may not only promote the levels of gene and protein expression of AMPK and GLUT4, but also initiate other methods of energy synthesis in order to meet energy requirements and avoid severe hypoglycemia during vigorous exercise. Furthermore, the enhanced expression of GLUT4 protein may improve glucose transport rates, thus increasing its utilization.

In conclusion, the antifatigue effect of *G. eucheumoides* was analyzed and the physiological and molecular mechanisms underlying this effect were investigated. The results demonstrated that *G. eucheumoides* exerts marked antifatigue effects, which may be achieved by regulating antifatigue-associated genes and increasing LDH concentration. Further studies are required to investigate the mechanims underlying these properties of *G. eucheumoides*.

## Figures and Tables

**Table I:** Primer sequences of target and reference genes used for quantitative polymerase chain reaction analyses.

Primer	Direction	Sequence (5′-3′)
GLUT4	Forward	5′-TCGTGGCCATATTTGGCTTTGTGG-3′
	Reverse	5′-TAAGGACCCATAGCATCCGCAACA-3′
AMPK	Forward	5′-TGACCGGACATAAAGTGGCTGTGA-3′
	Reverse	5′-TGATGATGTGAGGGTGCCTGAACA-3′
18sRNA	Forward	5′-CCTGGATACCGCAGCTAGGA-3′
	Reverse	5′-GCGGCGCAATACGAATGCCCC-3′

GLUT4, glucose transport protein 4; AMPK, AMP-activated protein kinase.

**Table II:** Effect of *G. eucheumoides* extract on the loaded swimming time of mice.

Group	Dose (mg/kg)	Number of mice	Loaded swimming time (sec)	Increment rate of loaded swimming time (%)
Control	0	10	31.3±0.2	-
Low-dose	20	10	38.7±0.4	19.1^a^
Medium-dose	40	10	41.3±0.5	32.0^b^
High-dose	80	10	36.4±0.2	16.3^a^

The increment rate of loaded swimming time represents the time of a group compared with that of the control group.

a Significant difference between the treatment and control groups (P<0.05);

b highly significant difference between the treatment and control groups (P<0.01).

**Table III:** Analysis of liver and muscle glycogen, LC, LDH and blood glucose concentration in mice.

Group	Liver glycogen (g/100 g)	Muscle glycogen (g/100 g)	LDH (U/l)	LC concentration (mM)	Blood glucose concentration (g/l)
Control	1.25±0.02	0.55±0.00	55.3±0.30	18.6±0.10	0.83±0.10
Low-dose	1.43±0.04^a^	0.78±0.01^a^	76.9±0.40^a^	15.4±0.06^a^	1.10±0.06^a^
Medium-dose	1.86±0.00^b^	1.12±0.11^b^	90.3±0.50^b^	13.8±0.10^b^	1.38±0.10^b^
High-dose	1.64±0.03^a^	0.82±0.09^a^	94.2±0.20^b^	11.3±0.10^b^	1.13±0.10^b^

a Significant difference between the treatment and control groups (P<0.05);

b highly significant difference between the treatment and control groups (P<0.01).

LC, lactic acid; LDH, lactate dehydrogenase.
